# A non-randomized clinical trial to examine patients’ experiences and communication during telemonitoring of pacemakers after five years follow-up

**DOI:** 10.1371/journal.pone.0261158

**Published:** 2021-12-23

**Authors:** Daniel Catalan-Matamoros, Antonio Lopez-Villegas, Cesar Leal Costa, Rafael Bautista-Mesa, Emilio Robles-Musso, Patricia Rocamora Perez, Remedios Lopez-Liria

**Affiliations:** 1 Health Research Centre, University of Almeria, Almeria, Spain; 2 Department of Communication Studies, University Carlos III of Madrid, Madrid, Spain; 3 Social Involvement of Critical and Emergency Medicine, CTS-609 Research Group, Hospital de Poniente, El Ejido-Almeria, Spain; 4 Nursing Department, University of Murcia, Murcia, Spain; 5 Management Unit, Hospital de Poniente, El Ejido-Almeria, Spain; 6 Intensive Care Unit, Hospital de Poniente, El Ejido-Almeria, Spain; 7 Department of Nursing Science, Physiotherapy and Medicine, University of Almeria, Almeria, Spain; Danube Private University, AUSTRIA

## Abstract

Patients with pacemakers need regular follow-ups which are demanding. Telemonitoring for pacemaker can provide a new opportunity to avoid follow-up visits. On the other hand, in-person visits could help patients with pacemakers to cope better with the anxiety linked to their condition and maintain better communication with their doctors than simple remote control of their device status. Therefore, our objective was to analyze the experiences and communication comparing telemonitoring (TM) versus conventional monitoring (CM) of patients with pacemakers. A single-center, controlled, non-randomized, non-blinded clinical trial was designed. Data were collected five years after implantation in a cohort of 89 consecutive patients assigned to two different groups: TM and CM. The ‘Generic Short Patient Experiences Questionnaire’ (GS-PEQ) was used to assess patients’ experiences, and the Healthcare Communication Questionnaire (HCCQ) was used to measure the communication of patients with healthcare professionals. Additionally, an ad-hoc survey including items from the ‘Telehealth Patient Satisfaction Survey’ and a ‘costs survey’ was used. After five years, 55 patients completed the study (TM = 21; CM = 34). Participants’ mean (±SD) age was 81 (±6.47), and 31% were females. No differences in baseline characteristics between groups were found. The comparative analyses TM *versus* CM showed some significant differences. According to GS-PEQ, TM users received adequate information about their diagnosis or afflictions (p = .035) and the treatment was better adapted to their situation (p = .009). Both groups reported negative experiences regarding their involvement in their treatment decisions, the waiting time before admission, and perceived a low-benefit. According to HCCQ, the TM group experienced poorer consultation management by the healthcare provider (p = .041). Participants reported positive overall communication experiences. The study provides insights into the experiences and communication in PM monitoring services as well as specific areas where users reported negative experiences such as the consultation management by clinicians.

**Trial registration:** ClinicalTrials.gov NCT02234245.

## Introduction

In Spain, in conformity with other European countries, heart-related deaths are the leading cause of mortality, followed by cancer [1–3]. One of the most frequent health interventions involves implanting a pacemaker (PM), which is a commonly implantable medical device (IMD). It is placed inside the patient’s body surgically [4]. It is estimated that each year 1.25 million permanent pacemakers are implanted worldwide, and approximately 500,000 implants are performed in Europe from which approximately 37,000 in Spain [5, 6]. In this regard, pacemakers’ telemonitoring is considered a new eHealth technology that allows the remote follow-up of patients. Previous research on telemonitoring of pacemakers has shown positive results on health-related quality of life and cost-effectiveness [7–9].

Decision-makers, healthcare managers, and politicians believe eHealth is an essential approach for the future of health systems as it would enable more personalized healthcare services [10]. Supporters of new health technologies believe that it would help patients and that the data obtained shall be useful for clinicians. Recent studies mention that telemonitoring might certainly not be so well-regarded by patients [10–12]. These new technologies may bring some degree of ambiguity and uncertainty among patients with questions such as ‘is my health status ok?’, ‘is the device functioning properly?’, ‘does my clinician perform a proper remote follow-up?’

Consequently, the adaptability and acceptability of new technology systems to its users must not be taken for granted. The literature reports very few studies enquiring patients’ experiences in using the last generation of PM models with remote follow-up, especially relating to the communication in these new clinical environments when there is a severe health issue in a very aged population lacking eHealth literacy skills. These studies have shown how patients with remote follow-up received less information about their diagnosis/afflictions [13] and similar experiences regarding communication than the patients with hospital follow-up [14]. Other studies have assessed how PM users contact the clinicians, thus emphasizing that this communication particularly relates to technical concerns on the data transmission as well as the monitoring system [15]. It has been reported that users included in telecare interventions are usually dissatisfied [16].

According to international guidelines [17, 18] patients with PM should have an initial check up within 3–10 days after the implant, with a second visit taking place at 2–12 weeks. After this point, follow-up visits are recommended at 3–12 months intervals, depending on the PM type and clinical circumstances. All hospital visits should include an assessment of the patient’s clinical condition and device functioning, with readjustment or medication changes being made if determined necessary [7, 19]. According to the international consensus on the remote monitoring [18], PM specifications, and physicians’ criteria, the patients are asked to submit data at different moments. Since month 1, no visits are normally scheduled; however, the patients are called and referred to an in-office visit if data received detected any device dysfunction or cardiovascular event. The physicians have access to the device information by logging onto a password-protected website, and assess all remote transmissions. The number of visits to the hospital and/or transmissions from home per patient and year depended on the type of PM implanted and patient characteristics [9].

Patients with pacemakers live with anxiety, concerning their pacemaker and health [20]. These patients are considered “high-risk” as per the hospital settings since they experience severe illness. The hospital’s decreased communication in this new, remote follow-up approach may cause high emotional anxiety and uncertainty [21]. Therefore, telemonitoring of the pacemakers may involve higher doses of negative emotions than that in other settings. Any abnormality or doubts during PM telemonitoring requires quick action to prevent any cardiac events. For that reason, users have requested more precise feedbacks from their remote follow-up [13, 22]. This emphasizes that fluent, clear, and effective communication between clinicians and patients becomes even more relevant in these remote clinical environments. Therefore, our null hypothesis was as follows: “Patients following telemonitoring of pacemakers have negative perception about their experience and communication”. With these regards, this article aims to explore the patients’ experiences with pacemakers after five years follow-up, comparing telemonitoring *versus* conventional monitoring, while mainly focusing on the communication-related experience.

## Materials and methods

This study is a part of a larger project, The PONIENTE study, a controlled, single-center, non-randomized, or masked clinical trial in the Hospital de Poniente, El Ejido, Spain. The study compares a group of telemonitored PM users with another group following conventional monitoring, through a comprehensive assessment involving different perspectives like socioeconomic evaluation [23], effectiveness, and safety [24]. This project entails collaboration among chronic heart patients with a pacemaker, their relatives, cardiologists, nurses, psychologists, and health communication experts. The following protocol was approved by the Regional Ethics Committee for Health Research on 28 November 2012 (CEIC-AL: 53/2012). The study was developed in accordance with the Declaration of Helsinki and Spanish laws on data protection and patient rights. Each participant was informed verbally and in writing, signed the informed consent, and care was taken to maintain data privacy during the study and afterward. The study was also registered at ClinicalTrials.gov (ID number: NCT02234245). This paper presents the experiences and communication assessment of a long-term five-year follow-up reported by the participants in either group, starting from implantation (2012–2017).

**Fig 1:**
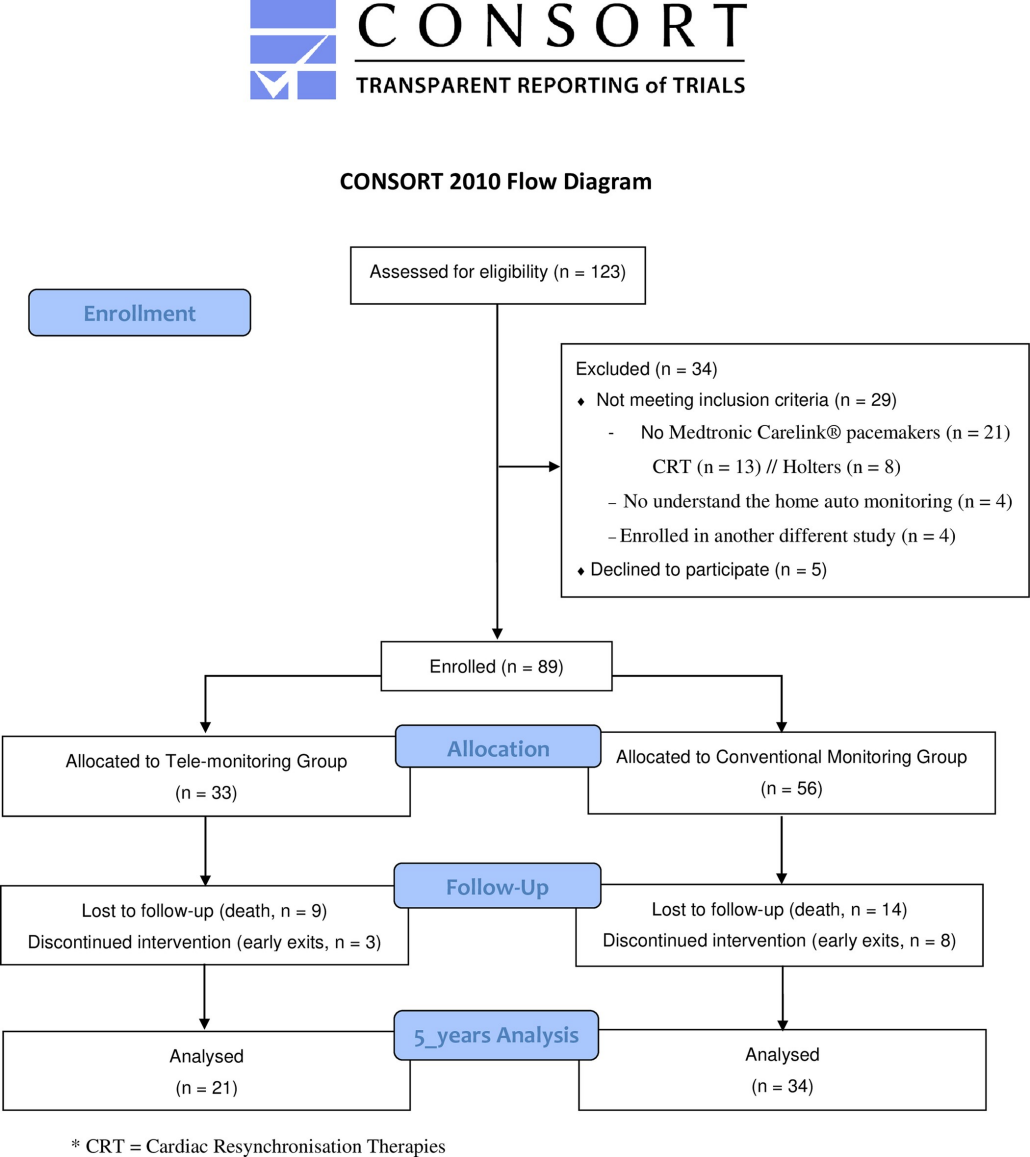
CONSORT flow diagram.

Participants were recruited through a convenience sampling method. The inclusion and exclusion criteria have recently been published in two papers [23, 24]. In total, 89 patients were eligible and invited to participate, of which 55 participated in the five-year follow-up. The mean (±SD) age was 81 (±6.47), and 31% of the participants were females. Fig 1 shows the Consort Flow Diagram. Among the 55 participants, 21 comprised the telemonitoring group, while 34 were included in the conventional follow-up group. The explicit nature of the intervention made it unfeasible to blind patients or clinicians to the group’s identity to which they had been located. Sample size was determined for the original project (the PONIENTE study) and it was based on the mean difference of the principal variable EQ5D. Assuming a two-tailed test with a significance level of 0.05 and a power of 0.80, a sample size of 45 patients per group was stablished as necessary for detecting a clinically relevant effect size d of 0.6 (effect size for reaching a mean difference of 0.12 points, with a standard deviation in each group of 0.20). Second, the questionnaire results were presented as a single question based on the comparison between the two groups, applying the Mann–Whitney U test for ordinal data and the chi-square test for nominal data and estimating the corresponding effect sizes measures (with 95% confidence interval): rank-biserial correlation and Cramer´s V respectively. For interpreting the magnitude of effect sizes, Lovakov and Agadullina (2021) rules are followed: r < 0.12—Very small; 0.12 < = r < 0.24 –Small; 0.24 < = r < 0.41 –Moderate; and r > = 0.41 –Large.

One month following PM implantation, all patients had a scheduled visit with a cardiologist where characteristics, advantages and disadvantages of both monitoring modalities were explained and offered for their selection [7]. In the case of patients chose the TM alternative, the cardiologist: 1) programmed the corresponding PM parameters; 2) explained to the patients the use of Monitor Carelink and the protocol to perform after sending data was required and 3) requested the service from the supplier company. In accordance with international guidelines [17, 18], PM specifications and physician criteria, patients were asked to submit data at different times. No visits were scheduled in the TM group. Pacemakers used in this study did not alert to patients to visit the hospital if a cardiovascular event was detected, however they were phoned and referred for a hospital visit if the data received detected a device dysfunction or a cardiac event. Patients assigned to the CM group were managed according to the standard practices of the Poniente Hospital, with follow-up visits scheduled according to physician criterion. Patients were interviewed on the date of implantation (physically in the hospital) and 60 months after pacemaker implantation (by phone). At each scheduled visit and upon completion of the study, the research team revised the medical record for identifying cardiovascular events, changes in patient management and PM reprogramming [7].

Data were collected at 60 months after pacemaker implantation. Evaluation of the participants in either group was done based on an *ad-hoc* questionnaire developed through two validated tools. The questionnaire comprised the full version of the Generic Short Patient Experiences Questionnaire (GS-PEQ) [25], and the adapted version of the HCCQ (Health Care Communication Questionnaire) [26]. Table 1 represents the *ad-hoc* questionnaire. The GS-PEQ is a brief instrument assessing patients’ experiences with hospital care (see items 1–10). The HCCQ aims to measure outpatients’ communication experience with hospital healthcare professionals (see items 11–17).

**Table 1:** Questionnaire details.

Items	Possible answers
1. Did the clinicians talk to you in a way that was easy to understand?	1 = Not at all; 2 = To a small extent; 3 = To a moderate extent; 4 = To a large extent; 5 = To a very large extent
2. Do you have confidence in the clinicians’ professional skills?	1 = Not at all; 2 = To a small extent; 3 = To a moderate extent; 4 = To a large extent; 5 = To a very large extent
3. Did you get sufficient information about your diagnosis/afflictions?	1 = Not at all; 2 = To a small extent; 3 = To a moderate extent; 4 = To a large extent; 5 = To a very large extent
4. Did you perceive the treatment as adapted to your situation?	1 = Not at all; 2 = To a small extent; 3 = To a moderate extent; 4 = To a large extent; 5 = To a very large extent
5. Were you involved in decisions regarding your treatment?	1 = Not at all; 2 = To a small extent; 3 = To a moderate extent; 4 = To a large extent; 5 = To a very large extent
6. Did you perceive the institution’s work as well organised?	1 = Not at all; 2 = To a small extent; 3 = To a moderate extent; 4 = To a large extent; 5 = To a very large extent
7. Did you have to wait before you were admitted for services at the institution?	1 = Not at all; 2 = To a small extent; 3 = To a moderate extent; 4 = To a large extent; 5 = To a very large extent
8. Overall, was the help and treatment you received at the institution satisfactory?	1 = Not at all; 2 = To a small extent; 3 = To a moderate extent; 4 = To a large extent; 5 = To a very large extent
9. Overall, what benefit have you had from the care at the institution?	1 = Not at all; 2 = To a small extent; 3 = To a moderate extent; 4 = To a large extent; 5 = To a very large extent
10. Do you believe that you were in any way given incorrect treatment?	1 = Not at all; 2 = To a small extent; 3 = To a moderate extent; 4 = To a large extent; 5 = To a very large extent
11. I was asked questions in an aggressive manner	1 = Not at all; 2 = To a small extent; 3 = To a moderate extent; 4 = To a large extent; 5 = To a very large extent
12. I have been given answers in an aggressive manner	1 = Not at all; 2 = To a small extent; 3 = To a moderate extent; 4 = To a large extent; 5 = To a very large extent
13. I have been treated with kindness	1 = Not at all; 2 = To a small extent; 3 = To a moderate extent; 4 = To a large extent; 5 = To a very large extent
14. I have been treated in a rude and hasty manner	1 = Not at all; 2 = To a small extent; 3 = To a moderate extent; 4 = To a large extent; 5 = To a very large extent
15. The healthcare provider addressed me with a smile	1 = Not at all; 2 = To a small extent; 3 = To a moderate extent; 4 = To a large extent; 5 = To a very large extent
16. The healthcare provider was able to manage the consultation	1 = Not at all; 2 = To a small extent; 3 = To a moderate extent; 4 = To a large extent; 5 = To a very large extent
17. The healthcare provider showed respect for my privacy	1 = Not at all; 2 = To a small extent; 3 = To a moderate extent; 4 = To a large extent; 5 = To a very large extent
18. How many kilometres is your home from hospital?	Number of kilometres
19. How much time does it take you to attend a cardiology consultation?	1 = <1 hour; 2 = 1–2 hours; 3 = 2–3 hours
20. What type of transport do you use to travel to hospital?	1 = Public transport; 2 = Own car; 3 = Ambulance; 4 = Taxi; 5 = Other
21. Which is your labour situation now?	1 = Working; 2 = Unemployed; 3 = Pensioner; 4 = Sick leave; 5 = Other
22. Do you need to be accompanied by any relative or friend to attend the cardiology consultation at hospital?	1 = No; 2 = Yes
23. Which is the labour situation of your accompanying person?	1 = Working; 2 = Unemployed; 3 = Pensioner
24. Have you or the accompanying person any expenses when travelling to hospital?	1 = No; 2 = Yes
25. Budget expended.	In euros
26. How many times have you phoned the pacemakers office at hospital?	1 = None; 2 = Once; 3 = Twice; 4 = More than 2
27. How many times have you attended the emergency ward for a problem related to your pacemaker in either the hospital or primary healthcare centre?	1 = None; 2 = Once; 3 = Twice; 4 = More than 2

Additionally, an adapted version of the telehealth patient satisfaction survey [27] and the costs survey [28] were used to evaluate other aspects of the telehealth experience among patients with home-monitored pacemakers such as patients’ experiences with the home monitoring technology and some specific cost-related inquiries regarding the pacemaker monitoring. The questionnaire was obtained either in-hospital or over the telephone.

This study followed the same statistical analyses that have been described in a previous study [29]. Continuous variables were expressed as means with standard deviations (SDs), and categorical variables were presented as actual numbers and percentages. First, patient baseline characteristics and possible differences between groups were compared using a difference in the mean values by employing a test for continuous variables and a difference in proportions test (binomial method), or the Chi-square test (replaced by Fisher’s exact test for cells with n < 5 cases) for qualitative variables. Second, the questionnaire results were presented as a single question based on the comparison between the two groups, applying the Mann–Whitney U test for ordinal data and the chi-square test for nominal data. In addition, effect sizes (rank-biserial correlation r for Mann-Whitney U test, and Cramer´s V for tests based on contingency tables) and 95% confidence intervals are included. Analyses were carried out using a dedicated statistical software (SPSS, Version 24.0; IBM, Armonk, NY, USA)

## Results

Baseline characteristics, depending on the intervention status, telemonitoring or conventional monitoring, have been presented in Table 2. There was no significant difference between the telemonitoring and the conventional monitoring group regarding other clinical characteristics except for hospital visits and total transmissions. The most frequent pacing indication was ‘atrioventricular block’ (70.9%), with syncope (60%) as the disease manifestation. Most participants (94,5%) did not require further hospitalization after the implant during the following five years.

**Table 2:** Selected patient baseline characteristics by intervention status five years after implant.

	All	Groups		p
		Remote Monitoring	Conventional Monitoring	
Age (mean) ±SD	81.00 ±6.47	81.14 ±7.30	80.91 ±6.01	0.690
Women (%)	17 (30.91)	8 (38.10)	9 (26.47)	0.365
DASI (mean) ±SD	19.46 ±6.29	19.05 ±5.77	19.72 ±6.67	0.842
EQ5D utilities (mean) ±SD	0.73 ±0.37	0.68 ±0.39	0.77 ±0.36	0.232
EQ5D VAS (mean) ±SD	73.27 ±15.99	73.81 ±13.96	72.94 ±17.33	0.879
***Pacing indication (%)***				
Sinus node disease	11 (20.00)	3 (14.29)	8 (23.53)	0.493
Atrioventricular block	39 (70.91)	15 (71.43)	24 (70.59)	
Others	5 (9.09)	3 (14.29)	2 (5.88)	
***Disease manifestations (%)***				
Syncope	33 (60.00)	13 (61.90)	20 (58.82)	0.681
Dizziness	16 (29.09)	7 (33.33)	9 (26.47)	
Dyspnoea	3 (5.45)	0 (0)	3 (8.82)	
Angina	3 (5.45)	1 (4.76)	2 (5.88)	
***Service derived (%)***				
Emergencies	11 (20.00)	4 (19.05)	7 (20.59)	0.516
Cardiology	32 (58.18)	14 (66.67)	18 (52.94)	
Other service	12 (21.82)	3 (14.29)	9 (26.47)	
***Stimulation (%)***				
VDD	14 (25.45)	5 (23.81)	9 (26.47)	0.595
DDD	30 (54.55)	12 (57.14)	18 (52.94)	
VVI	8 (14.55)	4 (19.05)	4 (11.76)	
VVIR	3 (5.45)	0 (0)	3 (8.82)	
**AF Paroxistic episodes *(%)***				
Yes	26 (47.27)	14 (66.67)	12 (35.29)	0.024
No	29 (52.73)	7 (33.33)	22 (64.71)	
**AF episodes duration (mean)±SD**				
	2.62 ±1.55	2.57 ±1.65	2.67 ±1.50	0.829
**Ischemic cerebrovascular event *(%)***				
Yes	2 (3.64)	1 (4.76)	1 (2.94)	0.622
No	53 (96.36)	20 (95.24)	33 (97.06)	
**Anticoagulation *(%)***				
Yes	20 (36.36)	11 (52.38)	9 (26.47)	0.052
No	35 (63.64)	10 (47.62)	25 (73.53)	
**Hospitalisation causes *(%)***				
No hospitalisation	52 (94.55)	19 (90.48)	33 (97.06)	
Friedrich	1 (1.82)	1 (4.76)	0 (0.00)	0.323
PM electrode dislocation	1 (1.82)	1 (4.76)	0 (0.00)	
PM Fracture electrode	1 (1.82)	0 (0.00)	1 (2.94)	
**Hospitalisation days after PM implantation** (mean)±SD				
	0.13 ±0.70	0.95 ±0.30	0.15 ±0.86	0.322
**Hospital visits** (mean)±SD				
	6.58 ±2.74	4.38 ±2.62	7.94 ±1.79	< 0.001
**Home transmissions** (mean)±SD				
	2.53 ±3.41	6.62 ±1.72	---	
**Total transmissions (mean)±**SD				
	9.11 ±2.72	11 ±2.93	7.94 ±1.79	< 0.001

n = 55 (TM group: 21; CM group: 34). 95CI: 95% confidence interval of means or proportions. EQ5D: EuroQoL-5D; DASI: Duke Activity Status Index. SD: Standard Deviation; AF: Atrial Fibrillation; PM: Pacemakers; VAS: Visual Analogue Scale; Friedrich: Pacemaker wound infection. Source: López-Liria et al. 2020 [24].

A few significant differences were observed between the telemonitoring and the conventional monitoring groups in the following items (Table 3):

- To question 3, which inquired if participants received satisfactory information regarding their diagnosis/afflictions, the telemonitoring group reported having received a higher level of adequate information than the conventional monitoring group (p = 0.035), with a medium effect size of 0.314 but with lower limit of confidence interval indicating very small effect. In total, 57% of telemonitoring participants stated they were sufficiently informed ‘to a very large extent,’ 38% ‘to a large extent,’ and only 5% ‘to a small extent.’ However, the conventional monitoring group reported 32% participants expressed they were sufficiently informed ‘to a very large extent,’ 44% ‘to a large extent,’ 12% ‘to a moderate extent,’ 6% ‘to a small extent,’ and ‘not at all.’
- About question 4, which queried if the participants perceived the treatment as adapted to their situation, the telemonitoring group reported better perception, 81% choosing ‘to a very large extent’ option. Conversely, the conventional monitoring group only reported 44% of participants opting ‘to a very large extent,’ 41% ‘to a large extent,’ and 14% ‘to a moderate extent.’ The difference between the two groups was significant (p *=* 0.027), but the magnitude of the effect ranging from very small to large.
- Concerning question 16 which asked for details on whether the healthcare provider was able to manage the periodic clinical consultation, the telemonitoring group reported more inadequate perception as 19% reported ‘to a moderate extent,’ 33% ‘to a large extent,’ and 48% ‘to a very large extent.’ The conventional monitoring group reported higher scores, 62% of participants agreed ‘to a large extent,’ and 35% ‘to a moderate extent.’ Differences between the two groups were statistically significant (p *=* 0.041), with a small effect size.
- Lastly, another significant difference with a large effect size (p < 0.001) was noted with question 25, which focused on the participants’ budget to attend the clinical consultations. The telemonitoring was low-priced than conventional monitoring.

**Table 3:** Results derived from the questionnaire.

Question	Answering categories*	Telemonitoring group (n = 21)	Conventional monitoring group (n = 34)	p-value	Effect size	CIL	CIU	Interpretation
Question 1†	1 = Not at all; 2 = To a small extent; 3 = To a moderate extent; 4 = To a large extent; 5 = To a very large extent	5 (1, 5)	5 (2, 5)	0.840	0.029	-0.28	0.333	very small
Question 2†		5 (3, 5)	5 (4, 5)	0.511	-0.088	-0.385	0.225	very small
Question 3†		5 (2, 5)	4 (1, 5)	*0.035	0.314	0.008	0.566	medium
Question 4†		5 (3, 5)	5 (3, 5)	*0.027	0.37	0.071	0.608	medium
Question 5†		2 (1, 5)	4 (1, 5)	0.361	-0.144	-0.432	0.17	small
Question 6†		4 (3, 5)	4 (3, 5)	0.406	-0.118	-0.41	0.196	very small
Question 7†		5 (3, 5)	5 (3, 5)	0.729	0.05	-0.26	0.352	very small
Question 8†		1 (1, 3)	1 (1, 3)	0.366	-0.073	-0.371	0.239	very small
Question 9†		1 (1, 4)	1 (1, 4)	0.454	0.09	-0.223	0.386	very small
Question 10†		4 (1, 5)	5 (1, 5)	0.948	0.01	-0.298	0.316	very small
Question 11†		1 (1, 2)	1 (1, 5)	0.748	0.017	-0.291	0.322	very small
Question 12†		1 (1, 2)	1 (1, 1)	0.203	0.048	-0.263	0.349	very small
Question 13†		5 (4, 5)	5 (2, 5)	0.827	-0.025	-0.329	0.284	very small
Question 14†		1 (1, 1)	1 (1, 2)	0.431	-0.029	-0.333	0.28	very small
Question 15†		5 (2, 5)	5 (1, 5)	0.072	0.259	-0.052	0.524	medium
Question 16†		5 (2, 5)	5 (1, 5)	*0.041	-0.193	-0.472	0.121	small
Question 17†		5 (2, 5)	5 (1, 5)	0.656	0.062	-0.25	0.361	very small
Question 18†	Number of kilometres	17 (5, 44)	16 (5, 44)	0.657	-0.008	-0.314	0.299	very small
Question 19†	1 = <1 hour	11 (52.38)	24 (70.59)	0.238	0.196	-0.118	0.474	small
	2 = 1–2 hours	9 (42.86)	10 (29.41)					
	3 = 2–3 hours	1 (4.76)	0 (0.0)					
Question 20‡	1 = Public transport	3 (14.3)	12 (35.3)	0.212	-0.697	-0.827	-0.497	large
	2 = Own car	17 (80.9)	20 (58.8)					
	3 = Ambulance	0 (0.0)	0 (0.0)					
	4 = Taxi	1 (4.8)	2 (5.9)					
	5 = Other	0 (0.0)	0 (0.0)					
Question 21‡	1 = Working	0 (0.0)	1 (2.9)	0.622	0.061	0	1	very small
	2 = Unemployed	0 (0.0)	0 (0.0)					
	3 = Pensioner	20 (95.2)	33 (97.1)					
	4 = Sick leave	1 (4.8)	0 (0.0)					
	5 = Other	0 (0.0)	0 (0.0)					
Question 22‡	Yes	19 (90.5)	32 (94.1)	0.632	0	0	1	very small
	No	2 (9.5)	2 (5.9)					
Question 23‡	1 = Working	13 (68.42)	21 (65.63)	1.00	0	0	1	very small
	2 = Unemployed	1 (5.26)	1 (3.13)					
	3 = Pensioner	5 (26.32)	10 (31.25)					
Question 24‡	Yes	21 (100)	34 (100)	1.00	-0.546	-0.731	-0.288	large
	No	0	0					
Question 25‡	Budget expended	29.38 (5.80–80.82)	59.96 (14.50–178.32)	< 0.001	-0.038	-0.341	0.272	very small
Question 26†	None	7 (33.3)	15 (44.1)	0.642	0.125	-0.189	0.415	small
	Once	12 (57.1)	17 (50.0)					
	Twice	1 (4.8)	2 (5.9)					
	More than 2	1 (4.8)	0 (0.0)					
Question 27†	None	15 (71.4)	26 (76.5)	0.776	0.035	-0.275	0.338	very small
	Once	5 (23.8)	5 (14.7)					
	Twice	1 (4.8)	2 (5.9)					
	More than 2	0 (0.0)	1 (2.9)					

*For questions 1–10, the following scoring was used: 1, not at all; 2, to a small extent; 3, to some extent; 4, to a large extent; and 5, to a very large extent.

†Data presented as median (min., max.).

‡Data presented as total number (percentage). CIL and CIU representing lower and upper limit of 95% confidence interval for the effect size, and Interpretation indicating magnitude of the effect.

The remaining items did not show any significant differences between the two groups. Overall, the communication was experienced positively by both groups (Table 3). However, participants shared some negative experiences about their PM monitoring, with no significant differences between groups. These findings can be summed up as follows:

e Question 5 inquiring whether participants had been involved in their treatment decisions reported low levels in both groups with 53% answers between ‘not at all’ and ‘to a small extent’ by the telemonitoring group and 33% by the conventional monitoring group.
f Question 7 focused on the waiting time before the participants were admitted to the hospital. The majority of participants (86% in the telemonitoring and 88% in the conventional monitoring group) scored the waiting time as ‘to a very large extent’ or ‘to a large extent.’
g Question 8 investigating whether the help and treatment that participants received at the institution were satisfactory, participants from both the groups reported low levels by 95% of the telemonitoring group and 88% of the conventional monitoring group scoring ‘not at all.’
h Question 9, which asked for the benefit perception that the participants received through institutional care, the patients showed poor levels. The option ‘not at all’ was the most preferred among 71% of the telemonitoring group while in the hospital group, 79% of patients opted for this.
i In question 10, both groups reported negative scores, although the telemonitoring group had a slightly better degree of belief regarding the fact that in any way, they had received an incorrect intervention than the conventional group (43% versus 50% in ‘to a very large extent’).

## Discussion

The H0 can be confirmed with our study showing some key differences in relation to experiences and communication of participants with pacemakers following telemonitoring *versus* conventional monitoring. Some significant findings were observed in the study. First, regarding the type of follow-up, telemonitoring compared to conventional monitoring showed a) better information, b) better adaptability to treatment, and c) more inadequate telemonitoring consultation management by clinicians. Second, with the experiences of PM patients, the study pointed out some areas requiring further improvement, such as d) the need to involve patients in the treatment-related decision-making; e) long waiting time before admission; f) unsatisfactory treatment; g) low-benefit perception, and; h) the high level of belief that the intervention received was incorrect. On the other hand, for items related to communication, participants reported overall positive experiences.

About the general experiences, the negative scores by both groups of participants differ from a similar study [13] in Norway; although overall positive experiences by PM users, both telemonitoring and conventional monitoring, in the short-term (6 months after surgery) was done. Another difference between these two studies was in the information received from clinicians. While the present study showed that telemonitoring patients received more sufficient information than the conventional monitoring group, the study from Norway found that telemonitoring participants received less sufficient information than conventional users. Previous studies conducted on cardiac implants patients suggest the need to deliver more information as there could be a higher prevalence of device malfunction, fear of death, and worries about maintaining themselves independently, driving, and sexual activities [30, 31].

Concerning the overall experience, the present study exhibited ‘not so positive’ experiences by patients. Some previous studies differ from this finding. In a recent assessment of new eHealth technologies in primary health care, participants reported that teleconsultation with clinicians was easy-to-use, flexible, and a convenient means of managing their health problems [32]. Other authors found that telemonitoring in implantable cardiac defibrillators appeared to be satisfactory for users [33]. The present study highlights that patients should be involved in decision-making, and the waiting time must be reduced. Other aspects of the experiences should also be considered, such as improving the patient’s perception in relation to PM monitoring benefits. With these regards, a study established that clinicians should present the benefits of PM monitoring to patients’ to improve awareness of the importance of such a process [34].

With the communication-related experiences, the participants reported positive experiences with no significant differences between the two groups. However, patients under remote follow-up of PM had less scheduled consultations at the hospital and fewer face-to-face contact with clinicians. After surgery, the telemonitoring group was informed that they would receive phone calls from the clinicians when something was not working well. Therefore, patients of this group were asked to assume that no news meant good news. The International consensus has confirmed this protocol on the monitoring of Cardiovascular Implantable Electronic Devices (CIED) [18]. The remote PM routinely gathers and sends data relating to the patient’s heart to the monitoring service center. Next, telemonitoring users were called up and invited to an in-hospital consultation when the information received showed any monitoring issue or heart event. Other authors recommend that healthcare professionals involved in eHealth services require good communication skills [35–37]. The present study showed that the patients reported positive communication experiences. Varma claimed that effective communication in telemonitoring requires the inclusion of a description of the benefits of this new monitoring model. Hence, the patient is aware of this new technology’s principles and safety [34]. During the communication, patients, along with their caregivers, need to know how to proceed, especially when there is a heart event or a PM issue, besides the expected reaction times. Additionally, to accomplish a sustainably effective communication and strengthen their relationship, the clinician should be familiar with the patient’s settings before calling in times of any emergency. Finally, our findings have also explored the economic perspective supporting previous studies that remote monitoring of older patients with pacemakers appears still as a cost-utility alternative to hospital monitoring after 5 years of follow-up [38].

This study presents many limitations that should be considered for an appropriate interpretation of the findings. Assessment of patients’ experiences and communication may entail feedback biases as the patients may deliberately respond to accomplish optimistic values. Even if an overall positive communication experience was observed, it might be related to the well-known ‘Hawthorne effect’ [39], which points out how participants could alter their behavior when involved in a study, and hence are receiving special attention and are eager to be glad for their clinicians. However, since the participants included in this study reported other negative general experiences beyond communication, it could be understood that the ‘Hawthorne effect’ might not be high. Another potential limitation lies in the data collection pattern, as the authors followed a retrospective method using a questionnaire that may be subject to recall bias. This can be avoided by the use of more reliable techniques like the recording of clinical encounters to assess communication between patients and healthcare providers in real-time [40]. One more critical limitation that must be highlighted is that the study is an open trial where all the members involved, including patients, researchers, and clinicians were aware of the monitoring. Finally, the low participant number is another relevant limitation. This 5-years follow-up after implant was not able to recruit more participants given the natural constrains of this study, considering the advanced age and the fragile health of this group of population. In addition, since the controversy on the usefulness of post hoc power analysis [41–43], we decided only to include the effect sizes (and 95% confidence interval) for the different comparisons. Due to the fact that both sample mean difference and sample variance are used as parameters in prospective power analysis, these parameters are highly unlikely to be close to their population values in small samples, so the sample power may be very different from the actual power. Moreover, sample size estimated in the original project was based on the principal variable and not on the individual items of the questionnaires. Regardless of these limitations, the authors believe that the PONIENTE project is a unique study providing long-term data in an area lacking research. As the study showed no differences between the telemonitoring and conventional monitoring groups in terms of health effectiveness and safety [24], and no significant differences were found concerning patient’s experiences and communication; telemonitoring may prove to be a good option for patients with difficulties in traveling to the hospital especially nowadays that healthcare services are shifting into virtual environments wherever possible. As remote healthcare services become a central solution in the current coronavirus pandemic, patients need to adopt these new technologies [44]. The present study provides critical data ready to be used for the implementation of new telemonitoring services. This would help overcome barriers in successfully implementing new eHealth services and models of care where patient’s experiences and communication are crucial for patient-centered care [45]. The eHealth services can thrive only when issues and concerns among each user are met and aligned, otherwise, the use of new technology would neither be feasible and implemented nor meaningful [16].

## Conclusion

With the increasing use of eHealth care services to meet the patients’ needs, the PONIENTE study provides vital data ready to be implemented in PM monitoring services, including both types of monitoring, remote and conventional. This study provides relevant insights into the experiences and communication, and although it confirms positive communication experiences, there is a need for improving specific areas where users reported negative experiences. All these actions would help develop better patient-centered eHealth services.

## Supporting information

S1 File (XLS)
