# Correction: A non-randomized clinical trial to examine patients’ experiences and communication during telemonitoring of pacemakers after five years follow-up

**DOI:** 10.1371/journal.pone.0265801

**Published:** 2022-03-16

**Authors:** Daniel Catalan-Matamoros, Antonio Lopez-Villegas, Cesar Leal Costa, Rafael Bautista-Mesa, Emilio Robles-Musso, Patricia Rocamora Perez, Remedios Lopez-Liria

There are errors in the Funding statement. The correct Funding statement is as follows: This study has been funded by Instituto de Salud Carlos III through the project "No. PI17/02056" (Co-funded by European Regional Development Fund/European Social Fund "A way to make Europe"/"Investing in your future"). The funding was awarded to AL-V, and the funders had no role in study design, data collection and analysis, decision to publish, or preparation of the manuscript.

## References

[1] Catalan-Matamoros D, Lopez-Villegas A, Leal Costa C, Bautista-Mesa R, Robles-Musso E, Rocamora Perez P, et al. (2021) A non-randomized clinical trial to examine patients’ experiences and communication during telemonitoring of pacemakers after five years follow-up. PLoS ONE 16(12): e0261158. 10.1371/journal.pone.0261158 34941904 PMC8699982

